# Impact of an e-learning module on personal protective equipment knowledge in student paramedics: a randomized controlled trial

**DOI:** 10.1186/s13756-020-00849-9

**Published:** 2020-11-10

**Authors:** Laurent Suppan, Loric Stuby, Birgit Gartner, Robert Larribau, Anne Iten, Mohamed Abbas, Stephan Harbarth, Mélanie Suppan

**Affiliations:** 1 Division of Emergency Medicine, Department of Anesthesiology, Clinical Pharmacology, Intensive Care and Emergency Medicine, University of Geneva Hospitals and Faculty of Medicine, Rue Gabrielle-Perret-Gentil 4, 1211 Geneva 14, Switzerland; 2 Genève TEAM Ambulances, Geneva, Switzerland; 3 Infection Control Program and WHO Collaborating Centre on Patient Safety, University of Geneva Hospitals and Faculty of Medicine, Geneva, Switzerland; 4 Division of Anesthesiology, Department of Anesthesiology, Clinical Pharmacology, Intensive Care and Emergency Medicine, University of Geneva Hospitals and Faculty of Medicine, Geneva, Switzerland

**Keywords:** Covid-19, e-learning, Personal protective equipment, Student paramedics, Emergency medical services, Randomized controlled trial

## Abstract

**Background:**

Prehospital professionals such as emergency physicians or paramedics must be able to choose and adequately don and doff personal protective equipment (PPE) in order to avoid COVID-19 infection. Our aim was to evaluate the impact of a gamified e-learning module on adequacy of PPE in student paramedics.

**Methods:**

This was a web-based, randomized 1:1, parallel-group, triple-blind controlled trial. Student paramedics from three Swiss schools were invited to participate. They were informed they would be presented with both an e-learning module and an abridged version of the current regional prehospital COVID-19 guidelines, albeit not in which order. After a set of 22 questions designed to assess baseline knowledge, the control group was shown the guidelines before answering a set of 14 post-intervention questions. The e-learning group was shown the gamified e-learning module right after the guidelines, and before answering post-intervention questions. The primary outcome was the difference in the percentage of adequate choices of PPE before and after the intervention.

**Results:**

The participation rate was of 71% (98/138). A total of 90 answer sets was analyzed. Adequate choice of PPE increased significantly both in the control (50% [33;83] vs 25% [25;50], *P* = .013) and in the e-learning group (67% [50;83] vs 25% [25;50], *P* = .001) following the intervention. Though the median of the difference was higher in the e-learning group, there was no statistically significant superiority over the control (33% [0;58] vs 17% [− 17;42], *P* = .087). The e-learning module was of greatest benefit in the subgroup of student paramedics who were actively working in an ambulance company (42% [8;58] vs 25% [− 17;42], *P* = 0.021). There was no significant effect in student paramedics who were not actively working in an ambulance service (0% [− 25;33] vs 17% [− 8;50], *P* = .584).

**Conclusions:**

The use of a gamified e-learning module increases the rate of adequate choice of PPE only among student paramedics actively working in an ambulance service. In this subgroup, combining this teaching modality with other interventions might help spare PPE and efficiently protect against COVID-19 infection.

## Background

The coronavirus disease 2019 (COVID-19) pandemic has strikingly increased the use of digital technologies [1, 2], and deployment of telemedicine interventions has been both hastened and amplified [3, 4]. Nevertheless, direct contact with patients is still necessary in situations such as critical emergencies [5]. Emergency medical services and prehospital professionals must therefore be prepared to deal with potentially infected patients [6].

Correctly choosing and adequately donning and doffing personal protective equipment (PPE) is essential to avoid infection. Both PPE and healthcare professionals are scarce resources that are essential to the management of the pandemic. Infection prevention and control (IPC) guidelines must therefore balance the need to spare PPE against the need of efficiently protecting healthcare professionals [7, 8]. Medical students are increasingly used on the frontline to overcome the shortage of certified physicians [9, 10]. Student paramedics who are not already actively working for an ambulance service might also be called upon. However, the ability of such students to correctly choose and use PPE might be limited [11].

A gamified e-learning module designed to help improve the adequate choice of PPE by prehospital professionals has previously been evaluated but failed to improve adequate PPE choice compared to a simple reminder of the COVID-19 IPC guidelines [12]. It was hypothesized that this lack of effect could have resulted from the already high level of pre-intervention knowledge in the tested population, which was primarily composed of seasoned providers. Our aim was to evaluate the impact of this gamified e-learning module on student paramedics, in whom pre-intervention knowledge should be lower, and who might therefore most benefit from such an intervention.

## Methods

### Study design and participants

**Fig. 1:**
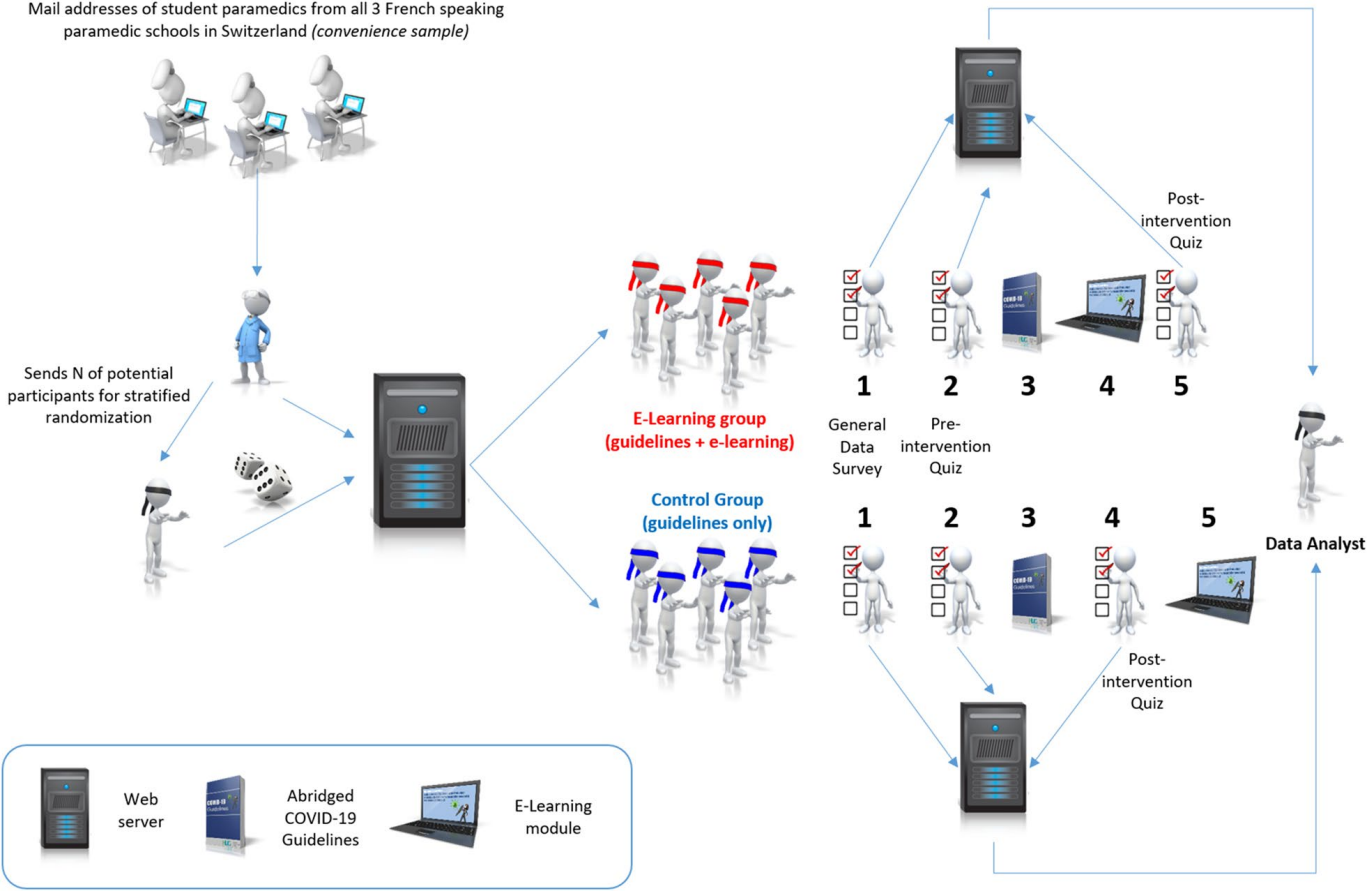
Study design, adapted from Suppan L. et al. [12]

This was a web-based, randomized 1:1, parallel-group, triple-blind controlled trial (Fig. 1) designed according to the CONSORT-EHEALTH statement [13], and incorporating relevant items from the CHERRIES guidelines [14]. A similar design has already been described and used previously [12].

In accordance to the International Committee of Medical Journal Editors, this trial was not registered as no health outcome was either assessed or recorded. A “Declaration of no objection” had already been issued by the regional ethics committee following a previously filed clarification of responsibility (Req-2020-00374) [12], thereby confirming that this kind of study design was not within the scope of the Swiss Federal Act on Research involving Human Beings [15].

There are three French-speaking paramedic schools in Switzerland, where student paramedics follow a 3-year curriculum before graduating. After approval by the paramedic instructors of the respective schools, we invited all students from the three schools, i.e. the whole target population, to participate in this study. Lists containing the e-mail addresses and study years of all students who had not participated in our first study were transmitted to one of the investigators (LSu). E-mail addresses were grouped by school and year of schooling before being sorted alphabetically. The number of potential participants for each of these nine groups was then sent to another investigator (MS) who performed a 1:1 randomization by group using computer generated charts. This investigator did not have access to any other information regarding the participants.

### Online platform and study sequence

A specific online platform was created under the Joomla 3.9 content management system (Open Source Matters, New York, USA). E-mail addresses were added to AcyMailing 5.1 distribution lists (Acyba, Lyon, France) according to their randomization allocation. The Community Survey Pro 5.4 component (CoreJoomla, Hyderabad, India) was used to create the study paths.

E-Mails inviting student paramedics to participate in the study were sent at the end of May 2020. These e-mails were identical except for the unique survey links which were automatically generated and contained specific tokens. No connection could be made between the tokens or the links and the e-mail addresses to ensure irreversible anonymization. The students were informed that the goal of the study was to assess their knowledge regarding the COVID-19 pandemic, and that both an e-learning module and an abridged version of the current COVID-19 guidelines would be presented. They were unaware that there would be two different study paths, and were told neither when nor in which order the learning materials would be presented.

Clicking on the survey link was considered as consent to participate in the study. The approximate time required to complete the study path was displayed both in the e-mail and on the online front page along with information regarding data protection and anonymization procedures. The students were informed that their answers could not be deleted once submitted as there was no way to identify a specific answer set. They were given the contacts of two investigators whom they could reach to ask further questions.

After agreeing to participate, the students were presented with a first set of 14 questions designed to gather demographic data and to determine how concerned they were about the COVID-19 pandemic. Baseline knowledge and self-confidence regarding the use of PPE were assessed by a second set of 22 questions, only four of which were specifically related to the choice of PPE. This rather high overall number of initial questions was designed to limit the priming effect [16], one of the potential shortcomings hypothesized in a previous publication [12]. These questions were followed by the intervention.

The control group was shown an abridged version of the current COVID-19 prehospital guidelines developed by the Geneva University Hospitals [17]. These guidelines were created under Visio 2013 (Microsoft Corporation, Redmond, USA) and extend over 12 pages. Flowcharts are used to describe the general management of COVID-19 patients and detail how PPE should be chosen according to the clinical situation. The full donning and doffing sequences are extensively described using numbered lists. Participants assigned to the control group proceeded to the last set of questions after having seen the guidelines. This set contained 14 questions, six of which were directly related to the choice of PPE, while the others assessed secondary outcomes such as PPE donning and doffing sequences. This latter outcome was assessed by asking the participants to put elements of each of these sequences in the correct order. Access to the e-learning module [18] was only granted once all the questions had been completed.

The e-learning group followed the gamified e-learning module right after seeing the abridged version of the guidelines. This interactive module was created under Articulate Storyline 3 (Articulate Global, New York, USA). Feedback [19], pretesting [16], avoiding content skipping [20, 21], embedded videos [22] and gamification [23] were used as learning mechanics. Gamification was used for donning and doffing sequences. To complete these sequences, the users were asked to drag pieces of PPE in the right order onto the photograph of an EMS provider. Chroma key technology [24] was used to remove the backgrounds from the photographs. This allowed us to update the photograph of the EMS provider each time a piece of protective equipment was dropped in the correct order, thereby giving an immediate and graphical feedback to the user.

Users were asked to rebuild these sequences for three different COVID-19 risk settings: procedures carrying a high-risk of COVID-19 transmission (i.e., intubation), suspected or confirmed COVID-19 with no need to perform a high-risk procedure, and no clinical suspicion of COVID-19. The whole module was designed to be completed in less than 15 min.

The post-intervention questions were displayed immediately upon completion of the e-learning module. All the original questions and their English translation can be seen in Additional file 1: Table S1. The questions used to assess the primary and secondary outcomes were identical to the ones used in a previous study on certified paramedics [12].

### Outcomes

The primary outcome was defined as the difference in the percentage of correct answers regarding the choice of PPE before and after the intervention. Four subgroup analyses were planned: by working status (whether the student paramedic was actively working in an ambulance service), by study year, by school, and by history of previous COVID infection.

Secondary outcomes were adequacy of both donning and doffing sequences, self-reported confidence in the participants’ ability of using PPE before and after the course, perceived usefulness of the learning path, and satisfaction as to the learning method.

### Data curation and statistical analysis

Data were extracted from the MySQL compatible database (MariaDB 5.5.5, MariaDB Foundation, Delaware, USA) to a comma-separated value file. Stata 15.1 (Stata Corporation, College Station, Texas, USA) was used for data curation and statistical analysis. Groups were renamed (Candor and Dauntless) and potentially revealing variables dropped to keep the data analyst (LSt) adequately blinded. Incomplete questionnaires, as well as those filled by students who had already seen the e-learning module or who were unable to access either the module or the guidelines, were excluded.

Continuous independent outcomes were assessed using Student’s *t* test or the Mann–Whitney U test depending on normality. Categorical outcomes were assessed using Fisher’s exact test. A two-sided *p* value less than 0.05 was considered significant. The normality of the distribution was first assessed graphically, then using the Shapiro–Wilk test. Continuous paired data were assessed using either the paired Student’s *t* test or the Wilcoxon matched-pairs signed-rank test depending on normality. The sign test for matched pairs was used if symmetry could not be proven. Categorical paired data were analyzed using asymptotic symmetry and marginal homogeneity tests.

Three pre-specified sensitivity analyses were conducted by excluding the participants who were unsatisfied by the course, those who responded false to a crucial multiple-choice question about understanding infection prevention (“Which of these measures is NOT one of the infection preventions measures?” False answer: systematic wearing of a double pair of gloves) and finally by excluding those who completed the path in more than 12 h.

### Data availability

The original data has been deposited to Mendeley Data [25].

## Results

**Fig. 2:**
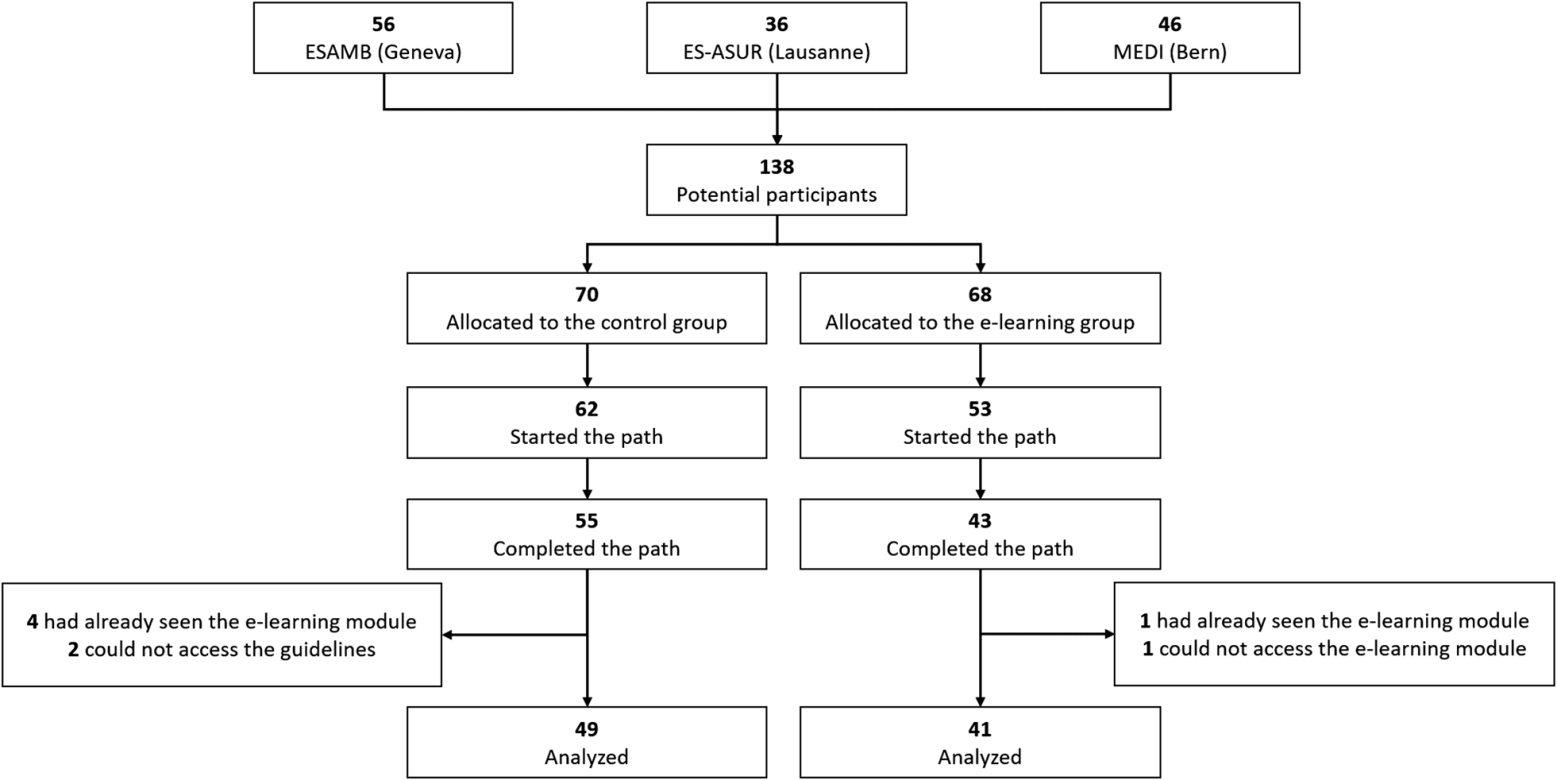
Study flowchart

Of the 138 potential participants, 98 (71%) completed the trial (Fig. 2). Ninety answer sets (65%) were analyzed after application of the exclusion criteria. The characteristics of the participants whose data was analyzed are described in Table 1.

**Table 1:** Characteristics of the study participants

	Control (n = 49)	E-Learning (n = 41)
Sex, female, n (%)	24 (49.0%)	16 (39.0%)
Age (years), median [Q1;Q3]	25 [23;30]	26 [24;30]
Study year, n (%)		
1	17 (34.7%)	12 (29.3%)
2	15 (30.6%)	12 (29.3%)
3	17 (34.7%)	17 (41.5%)
School, n (%)		
Bern (MEDI)	19 (38.8%)	15 (36.6%)
Geneva (ESAMB)	20 (40.8%)	15 (36.6%)
Lausanne (ES-ASUR)	10 (20.4%)	11 (26.8%)
Working canton, n (%)		
Bern	4 (8.2%)	3 (7.3%)
Fribourg	3 (6.1%)	0 (0.0%)
Geneva	4 (8.2%)	7 (17.1%)
Jura	3 (6.1%)	0 (0.0%)
Neuchatel	7 (14.3%)	5 (12.2%)
Valais	5 (10.2%)	6 (14.6%)
Vaud	9 (18.4%)	9 (22.0%)
Does not work	14 (28.6%)	11 (26.8%)
Previous general IPC course followed, n (%)	14 (28.6%)	13 (31.7%)
Previous COVID course followed, n (%)	10 (20.4%)	12 (29.3%)
History of COVID, n (%)	1 (2.0%)	1 (2.4%)
Previously seen HUG guidelines, n (%)	11 (22.4%)	11 (26.8%)

Totals may be unequal to 100% due to rounding

*IPC* infection prevention and control

Adequate choice of PPE significantly increased in both the control (*P* = 0.013) and the e-learning group (*P* = 0.001) after the intervention (Table 2). Though the median of the difference was higher in the e-learning group, there was no statistically significant superiority over the control (33% [0;58] versus 17% [− 17;42], *P* = 0.087).

**Table 2:** Change in proportion of adequate choice of personal protective equipment, with stratification according to working status

	Before	After	*P*
Overall			
Control (%), median [Q1;Q3]	25 [25;50]	50 [33;83]	.013
E-learning (%), median [Q1;Q3]	25 [25;50]	67 [50;83]	.001
Paramedic students not working in an ambulance service			
Control (%), median [Q1;Q3]	50 [25;50]	50 [33;83]	.180
E-Learning (%), median [Q1;Q3]	50 [25;75]	67 [33;83]	> .99
Paramedic students actively working in an ambulance service			
Control (%), median [Q1;Q3]	25 [25;50]	50 [33;67]	.058
E-learning (%), median [Q1;Q3]	25 [25;50]	67 [67;83]	< .001

**Fig. 3:**
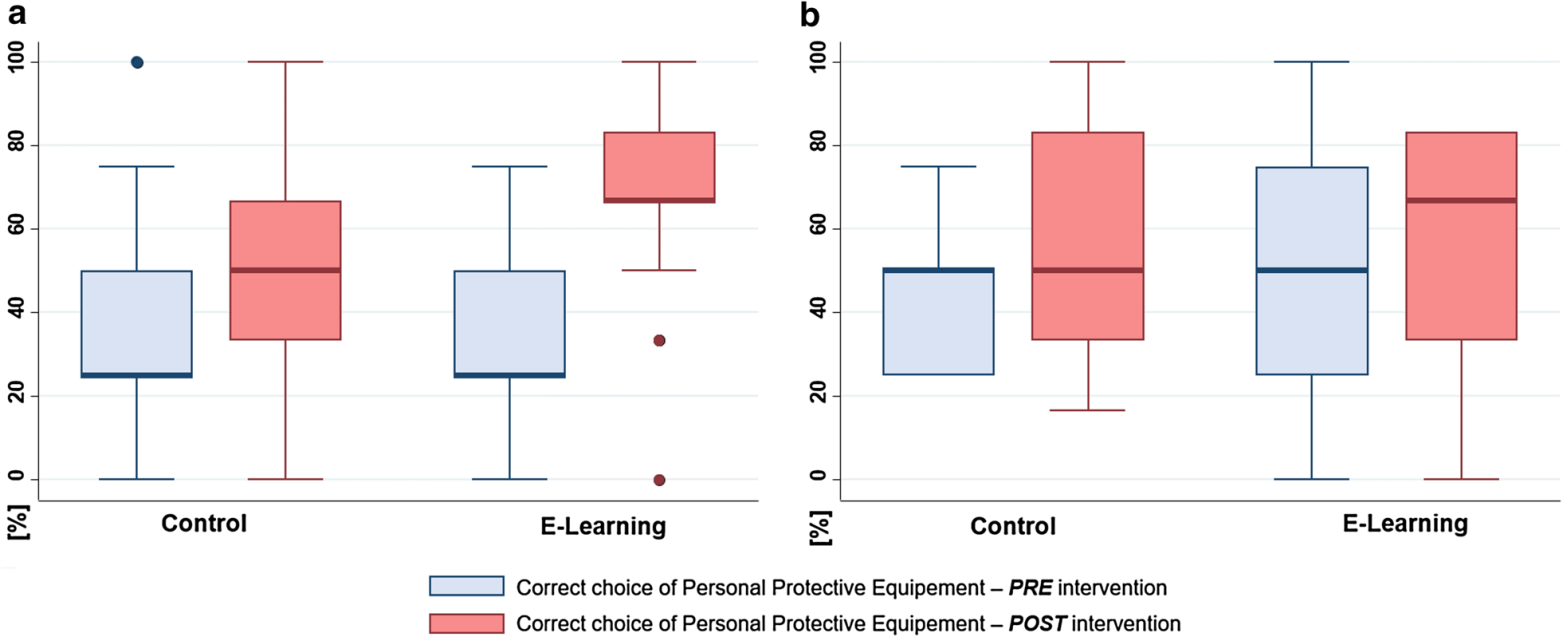
Adequate choice of personal protective equipment among **a** paramedic students actively working in an ambulance service and **b** paramedic students who were not working in an ambulance service

Stratification by working status showed that student paramedics who actively worked in an ambulance company had a significantly higher rate of correct answers in the e-learning group (Table 2; Fig. 3a). There was no significant difference among those who did not work in an ambulance service (Fig. 3b). The e-learning module yielded the best results among student paramedics who were schooled in Bern (*P* = 0.004) (Table 3). Detailed results of all the pre-planned subgroup analyses are presented in Table 3.

**Table 3:** Choice of personal protective equipment—detailed results

	Control (n = 49)	E-learning (n = 41)	*P*
Main outcome—difference in percentage of adequate choice of PPE (%), median [Q1;Q3]	17 [− 17;42]	33 [0;58]	.087
Adequate choice before (%), median [Q1;Q3]	25 [25;50]	25 [25;50]	.730
Adequate choice after (%), median [Q1;Q3]	50 [33;83]	67 [50;83]	.052
Main outcome by study year (%), median [Q1;Q3]			
1	25 [8;50]	33 [− 25;58]	.876
2	8 [− 25;33]	29 [− 4;42]	.161
3	17 [− 25;33]	42 [8;58]	.051
Main outcome by school (%), median [Q1;Q3]			
Bern (MEDI)	25 [− 25;42]	58 [25;67]	.004
Geneva (ESAMB)	13 [− 4;38]	0 [− 25;33]	.358
Lausanne (ES-ASUR)	29 [− 17;42]	42 [8;42]	.473
Main outcome by history of COVID (%), median [Q1;Q3]			
Never had COVID	21 [− 13;42]	33 [0;58]	.105
Had COVID	− 25 [− 25;− 25]	25 [25;25]	.317
Main outcome by working status (%), median [Q1;Q3]			
Actively works in an ambulance service	25 [− 17;42]	42 [8;58]	.021
Not working in an ambulance service	17 [− 8;50]	0 [− 25;33]	.584

*PPE* personal protective equipment

Donning and doffing (both assessed by the post-intervention quiz) sequences were in most cases incorrect. Correct answers regarding the donning sequence were only given by 7 participants (7.7% [95% CI 2.2–13.2]), with a similar rate of correct answers in the control group and in the e-learning group (*P* = 0.571). No participant was able to correctly describe the doffing sequence.

There was no difference in self-reported confidence in the ability of using PPE before and after the course (*P* = 0.521 in the control group and *P* = 0.666 in the e-learning group).

Most participants found the course at least “useful” (83.3% [95% CI 75.6–91.0]), with no statistically significant difference between groups (*P* = 0.167). Similarly, most participants were satisfied (80.0% at least “satisfied” [95% CI 71.7–88.3]), with no significant difference between groups (*P* = 0.731).

The effect was always in favour of the e-learning group in all three sensitivity analyses. The only sensitivity analysis to show a significant effect was the one excluding participants who took more than 12 h to complete the study path (*P* = 0.039). Excluding unsatisfied participants or those who disagreed with a crucial question about understanding infection prevention did not result in a significant change (*P* = 0.090 and *P* = 0.136, respectively).

Based on the observed change in adequate choice of PPE and using an alpha of 0.05, the estimated power for a two-sample means z test was of 31.4%.

## Discussion

Although the whole target population was invited to participate, and despite a relatively high participation rate, our sample size was limited and no significant effect on the difference in the percentage of correct answers regarding the choice of PPE was found overall. Nevertheless, the e-learning module significantly improved PPE choice among student paramedics who were actively engaged in clinical prehospital emergency work. Three main hypotheses might explain these findings. First, student paramedics actively working in ambulance services are usually either in their second or last school year. Their global knowledge should therefore be higher than that of their younger colleagues, and they probably therefore more readily grasp notions and subtleties which might be overlooked by less experienced students. Second, actively working in an ambulance service might lead student paramedics to use PPE more often, and to be better acquainted with the specificities of such equipment. Students with such knowledge and experience might reap the greatest benefit from this teaching modality as the gamified parts of the e-learning module specifically rely on the applying and understanding thinking skills described in Bloom’s revised taxonomy [26]. Finally, the baseline knowledge of student paramedics actively working in an ambulance service was lower than that of their colleagues who were not actively working. The reason for this lower level of knowledge is rather hard to fathom, but might be at least partly responsible for the significant effect found in this population.

Actively working in an ambulance service is probably not the only factor linked to the magnitude of the e-learning module’s impact. Stratification by school revealed that the students from the paramedic school in Bern benefitted the most from the use of the gamified e-learning module. These students were all part of the “actively working” subgroup as this school only accepts enrolment in a paramedic curriculum if candidates are already employed by an ambulance company. However, while participants from the ES-ASUR school in Lausanne were also actively working in an ambulance service, the e-learning did not yield as important an effect in this subgroup. Three main reasons might explain the differences found between schools. First, we must acknowledge a selection bias consecutive to our previous study, as many third year students schooled in Geneva were actively working in this city and were not included as they had already participated in a prior study [12]. Second, there was a West to East COVID contamination gradient in Switzerland, with a much higher contamination rate in the western regions. Therefore, prehospital professionals working in Geneva were more likely to have been exposed to COVID-19 patients than their colleagues from Bern. A lesser exposition to such patients and to related guidelines might allow students to approach teaching material without being prejudiced. Finally, differences in the teaching curricula between paramedic schools could be part of the explanation.

In this study, student paramedics fared no better than their more seasoned counterparts regarding their ability to correctly rebuild donning and doffing sequences. Indeed, a previous study has shown that only 4% of the certified paramedics were able to correctly rebuild the donning sequence, and that none of them was able to rebuild the doffing sequence [12]. The very high rate of incorrect answers might arise from different grounds. Our first hypothesis is that the online learning modalities might be ill-suited for the acquisition of such complex procedures when used alone. They might however prove useful if used in combination with other training modalities, such as live simulation, as e-learning modules have been shown to enhance practical skills acquisition [27]. Another hypothesis is that the sequence described in the learning materials might be ill-adapted to the actual prehospital field. This hypothesis could be tested by asking paramedics or student paramedics to actually perform the sequence described in either learning material during a live simulation session. Since we were unable to monitor whether the participants had actually followed the learning materials, we cannot rule out a lack of commitment on the part of the student paramedics. This hypothesis his however rather unlikely given the high level of satisfaction reported. Finally, the test method should also be questioned. However, the web platform used to ask participants to rebuild the sequences does not seem to be responsible for the high rate of incorrect answers. Indeed, the sequences initially displayed in the online quiz were left unchanged by only 4 participants, who were equally distributed between groups.

Apart from the limited sample size, this study has limitations that must be mentioned. Indeed, though we demonstrated a definite impact of the e-learning module in student paramedics who were actively working in an ambulance company, this result might not be reflected in the field. Moreover, owing to the design of this study, we were not able to determine whether this immediate result would be sustained over time. Further studies would be needed to ascertain both issues. Carrying out these studies would require a greater involvement on the part of the schools. Student paramedics could be randomized into two groups before answering the same sets of questions used in this study and finally attending a PPE workshop. Their performance regarding donning and doffing sequences could then be timed and assessed through the use of a standardized evaluation grid. The questionnaires and workshop could be repeated after 1 month to assess retention. The design of the guidelines used in this study could also lead to a bias. There are no federal prehospital guidelines in Switzerland, and we therefore decided to use those developed by the Geneva University Hospitals because they were the most comprehensive (if not the only) COVID-19 prehospital guidelines available in the French part of Switzerland at the time of this study. Moreover, the full version of these guidelines had already been used in a previous study carried out in a population of certified paramedics [12], thereby allowing a certain degree of comparison between the results. Finally, the inability of student paramedics to correctly rebuild both donning and doffing procedures shows that the use of a gamified e-learning module as an only training modality is insufficient. The impact of using this module in a multi-modal training session, including a live simulation, should be assessed.

## Conclusion

The use of a gamified e-learning module increases the rate of adequate choice of PPE only among student paramedics actively working in an ambulance service. In this subgroup, combining this teaching modality with other interventions might help spare PPE and efficiently protect against COVID-19 infection.

## Supplementary information

**Additional file 1**.

## Data Availability

The dataset generated and analyzed during the current study is available in the Mendeley Data repository, https://doi.org/10.17632/nks3nchhm4.1, https://data.mendeley.com/datasets/nks3nchhm4/1 [25]. The discussion section refers to previously published data, also available in the Mendeley Data repository, https://doi.org/10.17632/FFH678BV42.1, https://data.mendeley.com/datasets/ffh678bv42/1 [28]. The e-learning module can be viewed and downloaded freely (in both web and SCORM formats) from https://coronavirus.anesth.ch.

## Abbreviations

COVID-19: Coronavirus disease 2019
IPC: Infection prevention and control
PPE: Personal protective equipment
SARS-CoV-2: Severe Acute Respiratory Syndrome coronavirus 2

## References

[1] Ting DSW, Carin L, Dzau V, Wong TY (2020). Digital technology and COVID-19. Nat Med.

[2] Pérez Sust P, Solans O, Fajardo JC, Medina Peralta M, Rodenas P, Gabaldà J (2020). Turning the crisis into an opportunity: digital health strategies deployed during the COVID-19 outbreak. JMIR Public Health Surveill.

[3] Vilendrer S, Patel B, Chadwick W, Hwa M, Asch S, Pageler N, et al. Rapid deployment of inpatient telemedicine in response to COVID-19 across three health systems. J Am Med Inform Assoc. 2020; https://www.ncbi.nlm.nih.gov/pubmed/32495830. 10.1093/jamia/ocaa077 PMC7314045 32495830

[4] Mann DM, Chen J, Chunara R, Testa PA, Nov O. COVID-19 transforms health care through telemedicine: evidence from the field. J Am Med Inform Assoc. 2020; https://www.ncbi.nlm.nih.gov/pubmed/32324855. 10.1093/jamia/ocaa072 PMC7188161 32324855

[5] Holmes JL, Brake S, Docherty M, Lilford R, Watson S (2020). Emergency ambulance services for heart attack and stroke during UK’s COVID-19 lockdown. Lancet (Lond Engl).

[6] Ghazali DA, Ouersighni A, Gay M, Audebault V, Pavlovsky T, Casalino E (2020). Feedback to prepare EMS teams to manage infected patients with COVID-19: a case series. Prehosp Disaster Med..

[7] Lynch JB, Davitkov P, Anderson DJ, Bhimraj A, Cheng VC, Guzman-Cottrill J (2020). Infectious diseases society of America guidelines on infection prevention for health care personnel caring for patients with suspected or known COVID-19. Clin Infect Dis.

[8] Sommerstein R, Fux CA, Vuichard-Gysin D, Abbas M, Marschall J, Balmelli C (2020). Risk of SARS-CoV-2 transmission by aerosols, the rational use of masks, and protection of healthcare workers from COVID-19. Antimicrob Resist Infect Control.

[9] Klasen JM, Meienberg A, Nickel C, Bingisser R. SWAB team instead of SWAT team—students as front-line force during the COVID-19 pandemic. Med Educ. Blackwell Publishing Ltd; 2020. https://pubmed.ncbi.nlm.nih.gov/32403176/10.1111/medu.14224 PMC7272889 32403176

[10] Olum R, Kajjimu J, Kanyike AM, Chekwech G, Wekha G, Nassozi DR (2020). Perspective of medical students on the COVID-19 pandemic: survey of Nine Medical Schools in Uganda. JMIR Public Heal Surveill.

[11] Northington WE, Mahoney GM, Hahn ME, Suyama J, Hostler D (2007). Training retention of level C personal protective equipment use by emergency medical services personnel. Acad Emerg Med.

[12] Suppan L, Abbas M, Stuby L, Cottet P, Larribau R, Golay E (2020). Effect of an E-learning module on personal protective equipment proficiency by prehospital personnel: web-based, randomized controlled trial. J Med Internet Res..

[13] Eysenbach G, CONSORT-EHEALTH Group (2011). CONSORT-EHEALTH: improving and standardizing evaluation reports of web-based and mobile health interventions. J Med Internet Res..

[14] Eysenbach G (2004). Improving the quality of Web surveys: the checklist for reporting results of internet E-surveys (CHERRIES). J Med Internet Res.

[15] Swiss Confederation. CC 810.30 Federal Act of 30 September 2011 on research involving human beings (Human Research Act, HRA). 2011. https://www.admin.ch/opc/en/classified-compilation/20061313/index.html

[16] Latimier A, Riegert A, Peyre H, Ly ST, Casati R, Ramus F (2019). Does pre-testing promote better retention than post-testing?. NPJ Sci Learn.

[17] Brigade Sanitaire Cantonale. Coronavirus de la maladie COVID-19 : prise en charge préhospitalière [Internet]. Geneva; 2020. p. 1–17. https://smur.hug-ge.ch/sites/smur/files/guideline_covid19_1_11c.pdf

[18] Suppan M, Gartner B, Golay E, Stuby L, White M, Cottet P (2020). Teaching adequate prehospital use of personal protective equipment during the COVID-19 pandemic: development of a gamified e-learning module. JMIR Serious Games.

[19] Kopp V, Stark R, Fischer MR (2020). Fostering diagnostic knowledge through computer-supported, case-based worked examples: effects of erroneous examples and feedback. Med Educ.

[20] Raina S, Bernard L, Taylor B, Kaza S. Using eye-Tracking to investigate content skipping: a study on learning modules in cybersecurity. In: IEEE International conference on intelligence security informatics cybersecurity big data, ISI 2016. Institute of Electrical and Electronics Engineers Inc.; 2016. p. 261–6.

[21] Fouh E, Breakiron DA, Hamouda S, Farghally MF, Shaffer CA (2014). Exploring students learning behavior with an interactive etextbook in computer science courses. Comput Human Behav.

[22] Koka A, Suppan L, Cottet P, Carrera E, Stuby L, Suppan M (2020). Teaching NIHSS to paramedics, e-learning vs video: a randomized controlled trial. J Med Internet Res.

[23] Gentry SV, Gauthier A, Ehrstrom BLE, Wortley D, Lilienthal A, Car LT (2019). Serious gaming and gamification education in health professions: systematic review. J Med Internet Res.

[24] Bakkay MC, Pizenberg M, Carlier A, Balavoine E, Morin G, Charvillat V (2019). Protocols and software for simplified educational video capture and editing. J Comput Educ.

[25] Suppan L, Stuby L, Suppan M (2020). COVID-19—student paramedics PPE dataset..

[26] Krathwohl DR (2002). A revision of bloom’s taxonomy: an overview. Theory Pract.

[27] Li S, Li G, Liu Y, Xu W, Yang N, Chen H (2020). Development and assessment of a gastroscopy electronic learning system for primary learners: randomized controlled trial. J Med Internet Res.

[28] Suppan M, Stuby L, Abbas M, Gartner BA, Suppan L (2020). COVID-19—prehospital PPE dataset.

