# Supplementary material for: Impact of an e-learning module on personal protective equipment knowledge in student paramedics: a randomized controlled trial
Source: Antimicrob Resist Infect Control. 2020 Nov 10;9:185. doi: 10.1186/s13756-020-00849-9 (PMC7652675; doi:10.1186/s13756-020-00849-9)
Supplement: Supplementary file 1 — Additional file 1. [file 13756_2020_849_MOESM1_ESM.pdf]

Supplementary Table 1

Original study questions (in French) and English translation

Original questions (in French)		English translation	
Vous êtes:		You are:	
	Un homme<br>Une femme		A man<br>A woman
Quel est votre âge?		How old are you?	
	Saisie libre de 2 chiffres (validation Regex)		Free entry of 2 digits (Regex validation)
En quelle année d'étude êtes-vous?		What year are you in?	
	1<br>2<br>3<br>Autre		1<br>2<br>3<br>Other
Vous êtes étudiant à:		You are a student at:	
	Genève (ESAMB)<br>Lausanne (ES-ASUR)<br>Berne (MEDI)		Geneva (ESAMB)<br>Lausanne (ES-ASUR)<br>Bern (MEDI)
Travaillez-vous dans un service d'ambulance en parallèle de vos études?		Do you work in an ambulance service at the same time as your studies?	
	Oui<br>Non		Yes<br>No
Sur quel canton (principalement)?		In which canton (mainly)?	
	Berne<br>Fribourg<br>Genève<br>Jura<br>Neuchâtel<br>Valais<br>Vaud		Bern<br>Fribourg<br>Geneva<br>Jura<br>Neuchâtel<br>Valais<br>Vaud
Avez-vous déjà suivi un cours sur la prévention et le contrôle de l'infection (PCI)?		Have you ever taken an infection prevention and control (IPC) course?	
	Oui<br>Non		Yes<br>No
Quel est votre statut COVID?		What is your COVID status?	
	COVID négatif / pas testé<br>COVID positif - en isolement actuellement<br>COVID positif - guéri et non isolé<br>Je ne souhaite pas répondre à cette question		COVID negative / not tested<br>COVID positive - currently in isolation<br>COVID positive - cured and not isolated<br>I do not wish to answer this question
Avez-vous déjà vu le guideline COVID préhospitalier des HUG?		Have you ever seen the HUG prehospital COVID guideline?	
	Oui<br>Non		Yes<br>No

The questions used to compute the percentage of correct answers regarding the choice of personal protective equipment are highlighted in yellow. The expected answers are in bold and green.

**Original questions (in French)**	**English translation**
Avez-vous eu une formation spécifique relative au COVID?	Have you followed a COVID specific training session?
Oui<br>Non	Yes<br>No
Sous quelle forme cette formation vous a-t-elle été proposée?	How was this training session delivered?
Information par courrier électronique<br>Webinar<br>E-Learning<br>Autre formation en ligne<br>Annonce orale<br>Formation présentielle<br>Visioconférence<br>Atelier pratique<br>Vidéo<br>Autre	Information by email<br>Webinar<br>E-Learning<br>Other online training<br>Oral announcement<br>Face-to-face training session<br>Videoconferencing<br>Practical workshop<br>Video<br>Other
Vous sentez-vous confiant dans vos connaissances d'utilisation des équipements de protection individuels?	Do you feel confident in your ability to use of personal protective equipment?
Pas du tout confiant<br>Peu confiant<br>Neutre / indéterminé<br>Assez confiant<br>Très confiant	Not confident at all<br>Not confident<br>Neutral / undetermined<br>Fairly confident<br>Very confident
Les termes SARS-CoV-2 et COVID-19 sont interchangeables	The terms SARS-CoV-2 and COVID-19 are interchangeable
Oui<br>Non	Yes<br>No
Après avoir été infecté, les symptômes se déclarent généralement (chez la majorité des patients) après:	After becoming infected, symptoms usually appear (in the majority of patients) after:
Moins de 24 heures<br>1-2 jours<br>3-4 jours<br>5-6 jours<br>7-8 jours<br>9-10 jours<br>11 jours ou plus	Less than 24 hours<br>1-2 days<br>3-4 days<br>5-6 days<br>7-8 days<br>9-10 days<br>11 days or more
Une infection à SARS-CoV-2 est toujours symptomatique	SARS-CoV-2 infection is always symptomatic
Vrai<br>Faux	True<br>False
Seuls les patients âgés, ou souffrant de comorbidités importantes, vont présenter des complications graves pouvant aller jusqu'au décès	Only elderly patients, or patients suffering from significant comorbidities, will present serious complications which can lead to death.
Vrai<br>Faux	Vrai<br>Faux

The questions used to compute the percentage of correct answers regarding the choice of personal protective equipment are highlighted in yellow. The expected answers are in bold and green.

Original questions (in French)		English translation	
La transmission peut se faire par (plusieurs réponses possibles):		Transmission can be happen through (multiple answer question):	
	Contact direct avec le patient<br>Contact avec des surfaces touchées par le patient<br>Gouttelettes (éternuements, postillons)<br>Par aérosolisation / nébulisation		Direct contact with the patient<br>Contact with surfaces touched by the patient<br>Droplets (sneezing, …)<br>By aerosolization / nebulization
Vignette clinique : Il est 5 heures du matin. Vous êtes engagés par la CASU-144 pour un homme de 81 ans, connu pour hypertension artérielle, présentant une dyspnée brutale peu avant l'appel. Aucune autre information n'est disponible initialement. Le SMUR est indisponible. Le patient est à son domicile, au 2ème étage d'un immeuble en ville.		***Clinical scenario:*** It's 5 a.m. The emergency medical call center sends you on a mission to assess an 81 year old man known to have high blood pressure. He complains of a sudden-onset dyspnea that has started shortly before he called for help. No other information is initially available. No medical mobile unit is unavailable. The patient is at his home, on the 2nd floor of a building in town.	
Une tension artérielle élevée (systole > 180 mm Hg) peut-elle provoquer une dyspnée de ce type?		Can high blood pressure (systole> 180 mmHg) cause such dyspnea?	
	Oui<br>Non		Yes<br>No
En plus de l'équipement de protection standard, comment allez-vous vous équiper avant d'accéder au patient si vous êtes leader (plusieurs réponses possibles)?		In addition to the standard protective equipment, what other protective equipment will you don before accessing the patient if you are the leader (multiple answer question):	
	**Masque chirurgical**<br>Masque FFP-2<br>**Combinaison sans capuche**<br>Combinaison avec capuche<br>**Lunettes de protection**		**Surgical mask**<br>FFP2 mask<br>**Overall without hood**<br>Overall with hood<br>**Protective glasses**
En plus de l'équipement de protection standard, comment allez-vous vous équiper si vous êtes second et que vous restez à distance (plusieurs réponses possibles)?		In addition to the standard protective equipment, what other protective equipment will you don if you are assisting the leader and stay away from the patient (multiple answer question):	
	**Masque chirurgical**<br>Masque FFP-2<br>Combinaison sans capuche<br>Combinaison avec capuche<br>**Lunettes de protection**		**Surgical mask**<br>FFP2 mask<br>Coverall without hood<br>Coverall with hood<br>**Protective glasses**
Comment allez-vous oxygéner ce patient si la saturation est imprenable?		How are you going to oxygenate this patient if you cannot measure the oxygen saturation?	
	Pas d'oxygène à priori<br>Oxygène uniquement si cyanose<br>Oxygène aux lunettes, 2-4 litres / minute<br>Oxygène au masque "Venturi"<br>Oxygène au masque haute concentration (masque à réservoir)		No supplemental oxygen<br>Oxygen only if the patient is cyanotic<br>Oxygen through nasal cannula, 2-4 liters / minute<br>Oxygen with a "Venturi" mask<br>High-flow oxygen mask
La pose d'une voie veineuse périphérique (VVP) est-elle indiquée?		Should you place a peripheral venous line?	
	Oui<br>Non		Yes<br>No
Faudrait-il faire un ECG 12 dérivations dans cette situation?		Should you perform a 12 lead EKG?	
	Oui<br>Non		Yes<br>No

The questions used to compute the percentage of correct answers regarding the choice of personal protective equipment are highlighted in yellow. The expected answers are in bold and green. As we always expect prehospital personnel to wear protective goggles, this answer was always considered as correct, and is noted in bold and yellow.

Original questions (in French)	English translation
Vignette clinique : Vous intervenez en P1 pour état confusionnel aigu au domicile d'une femme de 80 ans, qui présente de la fièvre avec une toux sèche en aggravation depuis 6 jours. Depuis le pas de la porte vous constatez une FR>25/min.	***Clinical scenario:*** The emergency medical call center sends you to assess an 80-year-old woman who is confused, and has a fever with a dry cough that has worsened for 6 days. From the doorstep you notice a respiratory rate greater than 25 cycles / minute.
Quelles sont les causes possibles de cet état confusionnel (plusieurs réponses possibles)?	What are the possible causes of this confusion (multiple answer question):
Un globe urinaire<br>Un fécalome<br>Les médicaments habituels de la patiente<br>Une insuffisance rénale<br>Une consommation d'alcool<br>Une infection	An acute urinary retention<br>A fecaloma<br>The patient's usual medications<br>Kidney failure<br>Alcohol intoxication<br>An infection
Quel équipement de protection spécifique devez-vous mettre en tant que leader?	What other protective equipment will you don before accessing the patient if you are the leader (multiple choice question):
Masque chirurgical, combinaison sans capuchon, gants<br>Masque chirurgical, gants<br>**Masque FFP2, combinaison avec capuchon, lunettes, gants**<br>Masque FFP2, combinaison sans capuchon, lunettes, gants<br>Masque FFP2, surblouse, lunettes, gants	Surgical mask, coverall without hood, gloves<br>Surgical mask, gloves<br>**FFP2 mask, coverall with hood, goggles, gloves**<br>FFP2 mask, coverall without hood, goggles, gloves<br>FFP2 mask, protective gown, goggles, gloves
Quel EPP spécifique devez-vous mettre en tant que second si vous restez à distance?	What specific protective equipment should you don on as a second if you are assisting the leader and stay away from the patient (multiple choice question)?
Masque chirurgical, combinaison sans capuchon, gants<br>**Masque chirurgical**<br>Masque FFP2, combinaison avec capuchon, lunettes, gants<br>Masque FFP2, combinaison sans capuchon, lunettes, gants<br>Pas d'EPP spécifique si on respecte la distance > 3m	Surgical mask, coverall without hood, gloves<br>**Surgical mask**<br>FFP2 mask, coverall with hood, goggles, gloves<br>FFP2 mask, coverall without hood, goggles, gloves<br>No specific equipment if a distance of 3 meters or more is respected
Quels sont les tests indiqués dans cette situation?	Which diagnostic procedures should you perform in this situation?
Mesure de la glycémie capillaire<br>G-FAST<br>Test de Schellong<br>MMSE	Measurement of capillary blood glucose<br>G-FAST<br>Schellong test<br>MMSE
A priori, pensez-vous que le traitement de cette patiente pourra se faire de manière ambulatoire?	Do you think that the treatment of this patient can be done on an outpatient basis?
Oui<br>Non	Yes<br>No
Comment cette patiente doit-elle être installée sur le brancard?	How should this patient be placed on the stretcher?
En position couchée<br>En position semi-assise<br>Dans la position permettant le maximum de confort<br>Je ne la transporterais pas sur le brancard	Lying down<br>In a semi-sitting position<br>In the most comfortable position<br>I wouldn't carry her on the stretcher

The questions used to compute the percentage of correct answers regarding the choice of personal protective equipment are highlighted in yellow. The expected answers are in bold and green. As we always expect prehospital personnel to wear protective goggles, this answer was always considered as correct, and is noted in bold and yellow.

Original questions (in French)	English translation
Pensez-vous que le frottis à la recherche du virus responsable du COVID est fiable?	Do you think the test screening for the virus that causes COVID is reliable?
Oui, très fiable	Yes, very reliable
Oui, assez fiable	Yes, quite reliable
Neutre / indéterminé	Neutral / undetermined
Non, peu fiable	No, unreliable
Non, pas du tout fiable	No, not at all reliable
Après avoir eu le COVID-19, on est immunisé.	You are immune if you have already had COVID-19.
Vrai	True
Faux	False
Je ne sais pas	I do not know
La première vague de COVID semble passée. Une deuxième vague semble:	The first wave of COVID appears to be over. A second wave seems:
Très peu probable	Very unlikely
Peu probable	Unlikely
Neutre / indéterminé	Neutral / undetermined
Probable	Likely
Très probable	Very likely
A votre avis, une ventilation non-invasive (VNI) est-elle peut-elle être effectuée chez un patient suspect de COVID?	In your opinion, can non-invasive ventilation (NIV) be performed in a patient with suspected COVID?
Oui, cela ne change rien	Yes, it does not change anything
Oui, uniquement s'il s'agit d'un oedème aigu du poumon (OAP)	Yes, but only to treat acute pulmonary edema
Oui, uniquement s'il s'agit d'une BPCO décompensée	Yes, but only to treat acute COPD exacerbation
Non, la VNI devrait être évitée dans ce contexte	No, NIV should be avoided in this context

The questions used to compute the percentage of correct answers regarding the choice of personal protective equipment are highlighted in yellow. The expected answers are in bold and green. As we always expect prehospital personnel to wear protective goggles, this answer was always considered as correct, and is noted in bold and yellow.

The intervention (guidelines only vs. guidelines + e-learning) took place at this stage

Original questions (in French)	English translation
Vignette clinique : Il est 18 heures. Vous êtes engagés par la CASU-144 pour une détresse respiratoire avec suspicion de COVID-19 chez un patient de 27 ans, vivant à domicile avec sa compagne. Le patient et sa compagne ont tous deux été dépistés une semaine auparavant, car ils présentaient une toux sèche et un état fébrile. Le frottis du patient est revenu négatif, tandis que celui de sa compagne est revenu positif pour un SARS-CoV-2.	***Clinical scenario:*** It is 6:00 PM. The emergency medical call center sends you to assess a 27-year-old patient with respiratory distress and a suspicion of COVID-19. The patient is at home, where he lives with his partner. The patient and his partner were both screened a week ago because they had a dry cough and feverish state. The patient's smear came back negative, while his partner's came back positive for SARS-CoV-2.
Ce patient peut-il tout de même être atteint du COVID-19?	Can this patient have COVID-19?
Oui	Yes
Non	No
En plus de l'équipement de protection standard, comment allez-vous vous équiper avant d'accéder au patient si vous êtes leader (plusieurs réponses possibles)?	In addition to the standard protective equipment, what other protective equipment will you don before accessing the patient if you are the leader (multiple answer question):
**Masque chirurgical**	**Surgical mask**
Masque FFP-2	FFP2 mask
**Combinaison sans capuche**	**Overall without hood**
Combinaison avec capuche	Overall with hood
**Lunettes de protection**	**Protective glasses**

The questions used to compute the percentage of correct answers regarding the choice of personal protective equipment are highlighted in yellow. The expected answers are in bold and green. As we always expect prehospital personnel to wear protective goggles, this answer was always considered as correct, and is noted in bold and yellow.

Original questions (in French)	English translation
En plus de l'équipement de protection standard, comment allez-vous vous équiper si vous êtes second et que vous restez à distance (plusieurs réponses possibles)?	In addition to the standard protective equipment, what other protective equipment will you don if you are assisting the leader and stay away from the patient (multiple answer question):
**Masque chirurgical**<br>Masque FFP-2<br>Combinaison sans capuche<br>Combinaison avec capuche<br>**Lunettes de protection**	**Surgical mask**<br>FFP2 mask<br>Coverall without hood<br>Coverall with hood<br>**Protective glasses**
Quelle est la séquence à suivre si vous devez revêtir un équipement de protection individuel pour prendre soin d'un patient suspect de COVID en insuffisance respiratoire?	What is the sequence to follow if you need to put on personal protective equipment to care for a patient with respiratory failure who is COVID suspect?
Remettre dans l'ordre :<br>• Mise en place des lunettes de protection<br>• Désinfection des mains et mise d'une paire de gants non stériles<br>• Préparation d'une poubelle pour recueillir l'équipement en fin de déshabillage<br>• Mise en place du masque FFP-2 et test d'étanchéité<br>• Equipement avec une combinaison, capuche comprise	Reorder:<br>• Putting on protective goggles<br>• Hand disinfection and putting on a pair of non-sterile gloves<br>• Preparation of a trash can to collect the equipment at the end of the doffing procedure *(this element was not taken into account in the analysis)*<br>• Putting on the FFP-2 mask and performing a leakage test<br>• Equipment with coverall, hood included
Vignette clinique : Il est 15 heures. Vous êtes engagés sur un transport secondaire d'un patient en sepsis sévère depuis les urgences d'un centre cantonal pour un hôpital périphérique (frottis négatif). Le patient, un homme de 43 ans, a été admis pour des frissons présents depuis le matin même, précédés par une douleur lombaire gauche depuis 48 heures. Il est toujours hautement fébrile (température centrale: 40.2°C) et a été mis sous antibiotiques par voie intraveineuse. Il ne tousse pas, et ne pense pas avoir été en contact avec des personnes victimes du COVID.	***Clinical scenario:*** It is 3:00 PM. You are transferring a patient with severe sepsis from the emergency room of a university hospital to a regional hospital (COVID screen negative). The patient, a 43-year-old man, was admitted with chills that started this very morning, preceded by a left lower back pain that had been present for 48 hours. He still has high temperature (core temperature: 40.2 ° C) and has been put on intravenous antibiotics. He does not cough, and does not think he has been in contact with people infected by COVID.
En plus de l'équipement de protection standard, comment allez-vous vous équiper avant d'accéder au patient si vous êtes leader (plusieurs réponses possibles)?	In addition to the standard protective equipment, what other protective equipment will you don before accessing the patient if you are the leader (multiple answer question):
**Masque chirurgical**<br>Masque FFP-2<br>Combinaison sans capuche<br>Combinaison avec capuche<br>**Lunettes de protection**	**Surgical mask**<br>FFP2 mask<br>Overall without hood<br>Overall with hood<br>**Protective glasses**
En plus de l'équipement de protection standard, comment allez-vous vous équiper si vous êtes second et que vous restez à distance (plusieurs réponses possibles)?	In addition to the standard protective equipment, what other protective equipment will you don if you are assisting the leader and stay away from the patient (multiple answer question):
**Masque chirurgical**<br>Masque FFP-2<br>Combinaison sans capuche<br>Combinaison avec capuche<br>**Lunettes de protection**	**Surgical mask**<br>FFP2 mask<br>Coverall without hood<br>Coverall with hood<br>**Protective glasses**

The questions used to compute the percentage of correct answers regarding the choice of personal protective equipment are highlighted in yellow. The expected answers are in bold and green. As we always expect prehospital personnel to wear protective goggles, this answer was always considered as correct, and is noted in bold and yellow.

Original questions (in French)	English translation
Quelle séquence de déshabillage devez-vous suivre une fois la prise en charge de votre patient suspect de COVID terminée?	Which doffing sequence should you follow after having completed the management of a COVID victim?
Remettre dans l’ordre :<br>• Retirer le capuchon sans toucher les parties externes, puis retrousser progressivement la combinaison jusqu'aux chaussures<br>• Se désinfecter les mains et disposer des lunettes de protection<br>• Ouvrir la combinaison et se désinfecter les mains<br>• Retirer les chaussures, puis la combinaison<br>• Mise du matériel médical en contact avec le patient (stéthoscope, etc.) dans un sac prévu à cet effet<br>• Enlever les gants et se désinfecter les mains	Reorder:<br>• Remove the hood without touching the external parts, then gradually roll the corverall down to the shoes<br>• Disinfect your hands and put protective goggles in a separate bag<br>• Open the coverall and disinfect your hands<br>• Remove the shoes, then the coverall<br>• Put the medical equipment which was in contact with the patient (stethoscope, etc.) in a separate bag<br>• Remove the gloves and disinfect your hands
Parmi ces mesures, quelle est celle qui NE FAIT PAS partie des mesures de prévention de l'infection (une seule réponse possible)?	Which of these measures is NOT part of infection prevention measures (only one answer possible)?
Une technique de déshabillage maitrisée en enroulant la combinaison vers l’intérieur<br>Le port systématique d’une double couche de gants<br>L’hygiène des mains à l’aide d’une solution hydro-alcoolique<br>La désinfection des lunettes de protection à l’aide d’une compresse imbibée d’éthanol à 70%	A doffing technique which includes rolling the suit inwards<br>The systematic use of a double layer of gloves<br>Hand hygiene using a hydro-alcoholic solution<br>Disinfection of goggles using a swab soaked in 70% ethanol
Vignette clinique : Vous intervenez avec le SMUR au domicile d’une femme de 40 ans connue pour HTA, qui aurait présenté une dyspnée soudaine suivie d’une syncope. A votre arrivée sur site massage cardiaque en cours par un voisin de palier.	***Clinical scenario:*** You arrive at the same time as the medical mobile unit at the home of a 40-year-old woman known to have hypertension, who complained of sudden dyspnea followed by loss of consciousness. You immediately notice that the patient is in cardiac arrest. Chest compressions are provided by a neighbor.
Quel équipement de protection le leader doit-il porter?	What protective equipment should the leader wear? (multiple choice question)?
Masque chirurgical, combinaison sans capuchon, lunettes gants<br>**Masque FFP2, combinaison avec capuchon, lunettes, gants**<br>Masque FFP2, surblouse, lunettes, gants<br>Masque FFP2, lunettes, gants<br>Masque chirurgical, gants	Surgical mask, coverall without hood, glasses gloves<br>**FFP2 mask, coverall with hood, goggles, gloves**<br>FFP2 mask, protective gown, goggles, gloves<br>FFP2 mask, glasses, gloves<br>Surgical mask, gloves
Quel équipement de protection le médecin SMUR doit-il porter?	What protective equipment should the physician from the medical mobile unit wear? (multiple choice question)?
Masque chirurgical, combinaison sans capuchon, lunettes gants<br>**Masque FFP2, combinaison avec capuchon, lunettes, gants**<br>Masque FFP2, surblouse, lunettes, gants<br>Masque FFP2, lunettes, gants<br>Masque chirurgical, gants	Surgical mask, coverall without hood, glasses gloves<br>**FFP2 mask, coverall with hood, goggles, gloves**<br>FFP2 mask, protective gown, goggles, gloves<br>FFP2 mask, glasses, gloves<br>Surgical mask, gloves
Pour éviter de contracter le COVID, la phase la plus critique est:	To avoid COVID infection, the most critical phase is:
L'habillage<br>Le déshabillage<br>Ces phases sont aussi critiques l'une que l'autre	Donning<br>Doffing<br>These phases are equally critical

The questions used to compute the percentage of correct answers regarding the choice of personal protective equipment are highlighted in yellow. The expected answers are in bold and green. As we always expect prehospital personnel to wear protective goggles, this answer was always considered as correct, and is noted in bold and yellow.

Original questions (in French)		English translation	
Vous sentez-vous confiant dans vos connaissances d'utilisation des équipements de protection individuels?		Do you feel confident in your ability to use of personal protective equipment?	
	Pas du tout confiant<br>Peu confiant<br>Neutre / indéterminé<br>Assez confiant<br>Très confiant		Not confident at all<br>Not confident<br>Neutral / undetermined<br>Fairly confident<br>Very confident
Pensez-vous que cette formation vous a été utile / vous sera utile?		Do you think this training has been useful / will be useful to you?	
	Non, inutile<br>Non, peu utile<br>Ne sais pas / indéterminé<br>Oui, utile<br>Oui, très utile		No, not at all useful<br>No, not very useful<br>Don't know / undetermined<br>Yes, useful<br>Yes, very useful
Jusqu'à maintenant, à quel point êtes-vous satisfait·e de ce parcours de formation?		So far, how satisfied are you with this training course?	
	Très insatisfait<br>Insatisfait<br>Neutre / indécis<br>Satisfait<br>Très satisfait		Very unsatisfied<br>Dissatisfied<br>Neutral / undecided<br>Satisfied<br>Very satisfied

The questions used to compute the percentage of correct answers regarding the choice of personal protective equipment are highlighted in yellow. The expected answers are in bold and green. As we always expect prehospital personnel to wear protective goggles, this answer was always considered as correct, and is noted in bold and yellow.
